# Fluorescence and photophysical properties of xylene isomers in water: with experimental and theoretical approaches

**DOI:** 10.1098/rsos.171719

**Published:** 2018-02-07

**Authors:** Muhammad Farooq Saleem Khan, Jing Wu, Bo Liu, Cheng Cheng, Mona Akbar, Yidi Chai, Aisha Memon

**Affiliations:** State Key Joint Laboratory of Environment Simulation and Pollution Control, School of Environment, Tsinghua University, Beijing 100084, People's Republic of China

**Keywords:** fluorescence, excitation–emission, matrix, time-dependent density functional theory, xylene isomers

## Abstract

A thorough analysis of the photophysical properties involved in electronic transitions in excitation–emission spectra of xylene isomers has been carried out using the time-dependent density functional theory (PBEPBE/6-31 + G(d,p)) method. For the first time a structural and spectroscopic investigation to distinguish isomers of xylene, a widespread priority pollutant, was conducted experimentally and theoretically. The fluorescence properties of xylene isomers (sole and mixture (binary and ternary)) in water were studied. The fluorescence peak intensities of xylenes were linearly correlated to concentration, in the order of *p*-xylene > *o*-xylene > *m*-xylene at an excitation/emission wavelength (ex/em) of 260 nm/285 nm for *o*-, *m*-xylene and ex/em 265 nm/290 nm for *p*-xylene at the same concentration. The theoretical excitation/emission wavelengths were at ex/em 247 nm/267 nm, 248 nm/269 nm and 251 nm/307 nm for *o*-, *m*- and *p*-xylene, respectively. The vertical excitation and emission state energies of *p*-xylene (ex/em 4.94 eV/4.03 eV) were lower and the internal conversion energy difference (0.90 eV) was higher than those of *m*-xylene (ex/em 5.00 eV/4.60 eV) (0.4 eV) and *o*-xylene (ex/em 5.02 eV/4.64 eV) (0.377 eV). The order of theoretical emission and oscillator strength (0.0187 > 0.0175 > 0.0339) for *p*-xylene > *o*-xylene > *m*-xylene was observed to be in agreement with the experimental fluorescence intensities. These findings provide a novel fast method to distinguish isomers based on their photophysical properties.

## 1. Introduction

**Figure 1.**
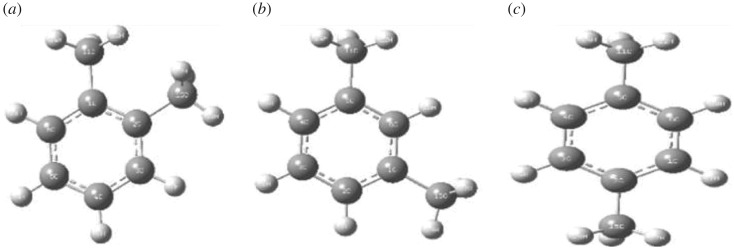
Molecular structures of (*a*) *o*-xylene, (*b*) *m*-xylene and (*c*) *p*-xylene.

Organic chemicals with benzene ring as the core constituent are among the widely available hazardous pollutants in our environment [1,2]. Contamination by organic compounds in a water environment is a serious threat to human health and lives [3,4]. Some volatile organic compounds, primarily single benzene ring compounds, such as benzene, toluene and xylenes [5], are widely found in the air [6], water [7–9] and soil [10]. Xylenes are one kind of aromatic hydrocarbons with a single benzene ring with two methyl groups attached in three isomeric forms, 1,2-dimethylbenzene, 1,3-dimethylbenzene and 1,4-dimethylbenzene, also called *o*-xylene, *m*-xylene and *p*-xylene, respectively [11] (figure 1). These isomers possess similar physico-chemical properties [12]. Xylenes are important industrial chemicals in the petroleum industry and are widely used organic solvents in several industrial processes [13,14]. Although the solubility of xylenes in water is very low, in the year 2015, around 1161 million tonnes of xylenes were produced in China. Therefore, xylenes are found in surface and groundwater [15]. The use of xylenes in large amounts tends to contaminate the surrounding environment and imparts dangerous effects on the environment [16] and human health; the toxicity ranges from LD_50_ = 1364 to LD_50_ = 5000 mg kg^−1^ [17–20]. The traditional measurements of these compounds are time-consuming and involve other chemicals, such as high-performance liquid chromatography [9]. There are various methods of detection and identification of xylenes, but few methods are effective to distinguish these isomers. Therefore, there arises an increasing demand to develop a fast analytical method like fluorescence spectroscopy [21] for their identification with less effort [22,23].

In recent years, computerized simulation has become an alternative method to facilitate the understanding of experimental data for the majority of scientific studies and applications [24]. The time-dependent density functional theory (TD-DFT) is widely used with a reasonable computational activity through Gaussian software [25] to study theoretical absorption spectra. The TD-DFT with an appropriate functional can yield the results of geometries and energies of molecules. The accuracy of theoretical measurements towards experimental data is higher for small molecules than for larger ones. To overcome this deficiency, one can use large basis sets or different hybrid functionals (PBEPBE) with more computational efforts by computer [26]. Most of the TD-DFT studies focus on absorption or excitation transition energies [27]. To our best knowledge, a few studies on the fluorescence of compounds in solution have been done with the help of TD-DFT in the past [27].

In this study, for the first time, fluorescence properties of xylene isomers in water were investigated with fluorescence spectroscopy and TD-DFT simulation. The electronic structure examined by fluorescence spectra and a quantum chemical method describes the interpretation of photophysical properties and also the direction of electronic density distribution, which determines the reactivity of each isomer in a complex water environment. The excited state and emission state geometries with possible solvent effects were examined with TD-DFT in conjunction with PBEPBE, whereas for the solvent effect the conductor-like polarizable continuum model (CPCM) was employed. The study of mixtures of different isomers provides information on the reactivity of isomers and their effect in aqueous environments. The fluorescence intensity and quantum chemistry information from experimental results and theoretical calculations provides understanding about molecular excitation and emission activity of xylenes in water. Fluorescence spectroscopy combined with TD-DFT calculations has provided an adequate understanding to describe photophysical properties of organic compounds with different isomers to identify and distinguish them in water.

## 2. Material and methods

### 2.1. Materials

Spectroscopic grade xylene isomers were purchased from AccuStandard (USA). Owing to high toxicity, these isomers were dissolved in methanol. Ultra-purified water was used to prepare all the solutions.

### 2.2. Sample preparation

Stock solutions (3.2 mg l^−1^) of the compounds were prepared by dissolving the exact amount of these compounds in purified water and were kept in brown glass bottles with a sealed cap; from these solutions different dilutions were prepared for further analysis. A mixture of two or three isomers was prepared in a ratio of 1 : 1 for binary mixture and 1 : 1 : 1 for ternary mixture in purified water. Stirring of the solution was carried out with a magnetic stirrer (SG–5402B) with an 8 × 15 mm stirring bar. All of the stock solutions were stored at 4°C prior to use.

### 2.3. Optical measurement

In this study, absorption spectra were recorded with a UV–visible spectrophotometer (UV-2401 PC, Hitachi, Japan) with a wavelength range from 190 to 800 nm. The fluorescence spectra were measured with a fluorescence spectrophotometer (F2700 Hitachi, Japan) in a cuvette with a side slit width of 5 nm and scan speed of 12 000. The excitation and emission wavelength ranges were set at 220 nm to 600 nm and 230 nm to 650 nm, respectively. The mean predicted value for fluorescence intensity of the mixture was calculated by the difference of the mixture data from the sole chemical data. The experiment was conducted at laboratory temperature (25°C). Fresh solutions were used for all measurements; emission spectra were not corrected for the spectral response of the instruments.

### 2.4. Quantum chemical calculations

The computational simulations were performed with the Gaussian 09 development program [28] (electronic supplementary material, S1). The ground and excited state geometries for each xylene isomer have been calculated by optimization with the 6-31 + G (d, p) basis set (double-zeta with diffuse functions on all non-hydrogen atoms and polarization on all atoms) using the PBEPBE functional [29–31]. The optimization and energy calculations were done in consideration of the solvent effect by using analytical gradient implementation with CPCM in Gaussian 09. For the confirmation of the stability of molecular structures, the vibrational spectra were calculated followed by each step. The vertical excitation energies of molecules were computed by using the TD-DFT [32] method for ground state geometries. Whereas the emission from the excited to the ground state was computed in a similar manner to optimization of the geometry from the previous excited state. The difference between the excited state energy and the fluorescence energy was considered to be the molecular interconversion energy. In the comparison of energy behaviour between molecules, this computational method provides comprehensive information. To study the computational fluorescence, the PBEPBE functional has been used in previous studies and could provide accurate theoretical results similar to experimental results [33]. In this work, all the calculations were performed by the PBEPBE functional with the 6-31 + G (d, P) basis set, and the solvent effect was examined by CPCM.

## 3. Results and discussion

### 3.1. Fluorescence properties of xylene isomers

**Figure 2.**
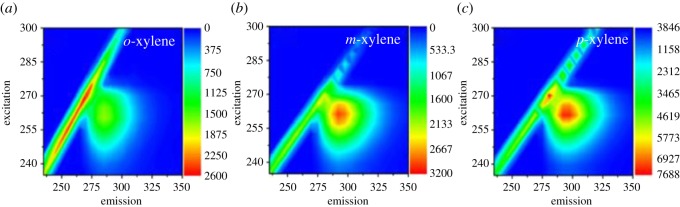
Fluorescence EEMs of (*a*) *o*-xylene, (*b*) *m*-xylene and (*c*) *p*-xylene.

**Figure 3.**
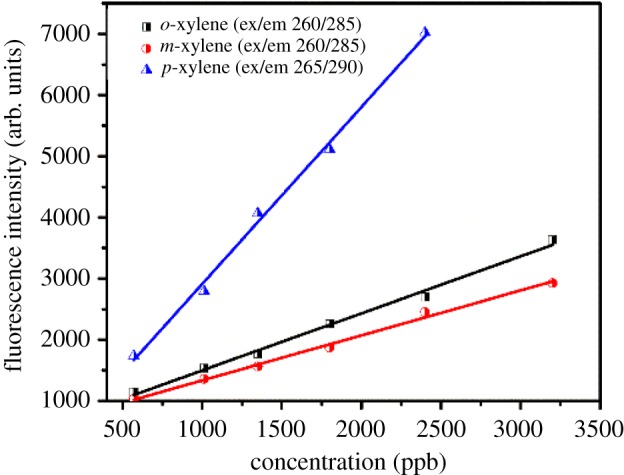
Fluorescence peak intensity versus concentration of xylene isomers.

Fluorescence spectroscopy is one of the emerging technologies to identify contamination in water bodies [34–36]. The fluorescence study of wastewater demonstrates that a sharp peak at an emission wavelength less than 380 nm is associated with single and double benzene rings, and an emission wavelength greater than 380 nm is associated with chemicals with polycyclic aromatic compounds [37,38]. The excitation–emission matrices (EEMs) of xylene isomers (*o*-, *m*- and *p*-xylene) at different concentrations in purified water were studied. There is only one peak around the excitation/emission wavelengths of 265/290 nm for each isomer as indicated in figure 2. The peak of *o*- and *m*-xylene is at an excitation wavelength of 260 nm and emission wavelength of 285 nm, whereas the excitation wavelength peak for *p*-xylene was found at 265 nm and the emission wavelength at 290 nm. Although the fluorescence excitation and emission wavelengths are almost the same for all three isomers with a minor difference of 5 nm, the fluorescence intensity of the peak was observed to be highest for *p*-xylene at the same concentration (figure 3). This indirectly shows that the order for fluorescence quantum yield of these isomers was *p*-xylene > *o*-xylene > *m*-xylene.

At higher concentrations, stronger intensity was found for all three xylene isomers in water, and fluorescence intensity exhibited a good linear correlation with concentration (figure 3). The correlation coefficients (*R*^2^) at ex/em 260 nm/285 nm for *o*- and *m*-xylene were and 0.997, respectively. Similarly, *p*-xylene has an *R*^2^ of 0.998 at 265 nm/290 nm. The difference of intensities is given below:

3.1 $$p\text{-xylene:}\;I=2.9c+18,\;R^{2}=0.998,$$

3.2 $$o\text{-xylene:}\;I=0.93c+566,\;R^{2}=0.997$$

3.3 $$m\text{-xylene:}\;I=0.73c+602,\;R^{2}=0.997,$$

where *I* is the fluorescence peak intensity (arb. units) and *c* is the concentration in µg l^−1^.

**Figure 4.**
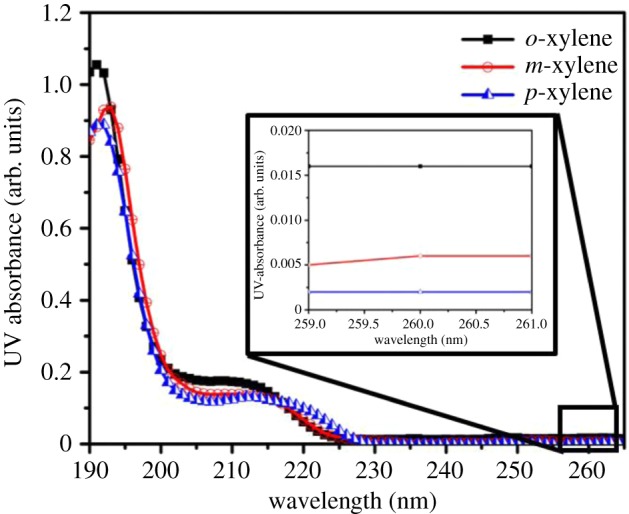
UV-absorbance of *o-*, *m-* and *p*-xylene in aqueous solution at a concentration of 3.2 mg l^−1^.

The study of the isomers with UV absorbance gives the highest value of absorbance at the wavelength 191–193 nm. The absorbance for *o*-xylene is greater than that for *m*-xylene and *p*-xylene at the same concentration (3.2 mg l^−1^) (figure 4). UV absorbance of xylene isomers reflects the difference in absorption [39] derived from the different structure of the molecules. The molar absorptivity (ε) was calculated to be 3.37 for *o*-xylene, 3.00 for *m*-xylene and 2.84 for *p*-xylene, by applying the Beer–Lambert law (equation (3.4)). The UV absorbance at an observed excitation wavelength of 260 nm shows the lowest value for *p*-xylene. These results indicate that *p*-xylene possesses the lowest molar absorptivity and therefore produces the highest quantum yield when compared with *o*- and *m*-xylenes.

3.4 $$\varepsilon =\frac{A}{lc},$$

where ε is the molecular absorptivity, *A* is the absorbance, *l* is the length of the light path and *c* is the concentration.

### 3.2. Fluorescence of binary and ternary mixtures

**Figure 5.**
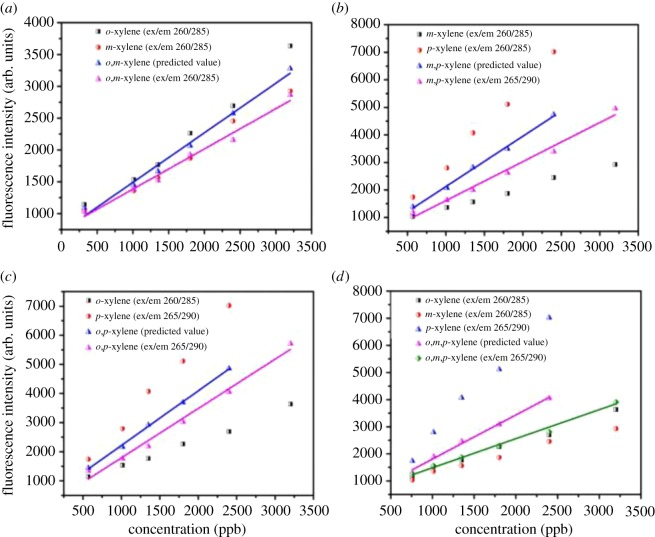
Fluorescence intensity at different concentrations of sole isomers versus binary and ternary mixtures: (*a*) sole isomers and binary mixture of *o*-, *m*-xylene; (*b*) sole isomers and binary mixture of *m*-,*p*-xylene; (*c*) sole isomers and binary mixture of *o*-,*p*-xylene; (*d*) ternary mixture with sole isomers.

The fluorescence properties have appeared to be unique for each isomer. The molecular geometry is the possible reason for the difference in fluorescence quantum yield. The location of the fluorescence peak of the mixture remains the same as that for sole chemicals; this indicates no significant shift in peak location. The fluorescence intensities of binary and ternary mixtures at different concentrations represent a similar trend. The fluorophores represent the fluorescence intensity in the tryptophan region for sole and mixture solutions [40]. When the same ratio (1 : 1) of isomers in a mixture was studied, the fluorescence intensity showed good linear correlation to the concentration. The fluorescence peak intensities of a binary combination of *o*- and *m*-xylene exhibit a significant difference and these intensities were found near to *m*-xylene in a good linear correlation to concentration with an *R*^2^ of 0.992. The difference in slope values between the mixture and sole *o*- and *m*-xylene (0.93 and 0.73) indicates an interaction between *o*- and *m*-xylene mixtures in aqueous solution. The fluorescence intensities of a binary mixture of *o*- and *p*-xylene showed a significant difference from the experimental data, which show the fluorescence intensity in the middle, measured from sole isomers, and there was good linear correlation to the concentration, with an *R*^2^ of 0.993 (figure 5). The fluorescence intensity of a binary mixture of *m*- and *p*-xylene showed a significant difference as compared to the fluorescence intensity of individual isomers, and there was a good linear correlation to concentration, with an *R*^2^ of 0.991. The fluorescence intensity of a ternary mixture of *o*-, *m*- and *p*-xylene showed a significant difference from the experimental data, which showed the fluorescence intensity near to that of *o*-xylene, measured from sole isomers, and there was good linear correlation to concentration, for an experimental *R*^2^ of 0.997.

The fluorescence study of binary mixtures with the same ratio has shown higher deviation in slope value (0.4) for *m*- and *p*-xylene than for *o*- and *m*-xylene (0.2) and *o*- and *p*-xylene (0.1); this shows a strong interaction between the *m*- and *p*-xylene binary mixture in water solution. The experimental interaction intensities of the binary and ternary mixtures are significantly lower than the predicted ones. This indicates that there exist some interactions between two or three isomers in the mixture. The difference between the experimental and predicted intensities of the ternary mixture was the highest and that of a binary mixture of *o*- and *m*-xylene was the lowest. This also indicates the existence of an interaction between the two isomers in solution.

The deviation in theoretical predicted and experimental value indicated in average percentages for the mixtures are 10% (*o*,*m*-xylene), 21% (*o*,*p*-xylene), 32% (*m*,*p*-xylene) and 27% (*o*,*m*,*p*-xylene). It appears from the percentage deviation values that the maximum molecular interaction has been observed for the *m*,*p*-xylene binary mixture and the lowest was observed for *o*,*m*-xylene. In the case of the ternary mixture, the percentage deviation was lower than that for *m*,*p*-xylene, but remains higher than those of other binary mixtures. The presence of *p*-xylene increases the intensity and molecular interaction hence generates an impact on fluorescence intensity with other molecules in different mixtures.

### 3.3. Computational quantum mechanics

**Figure 6.**
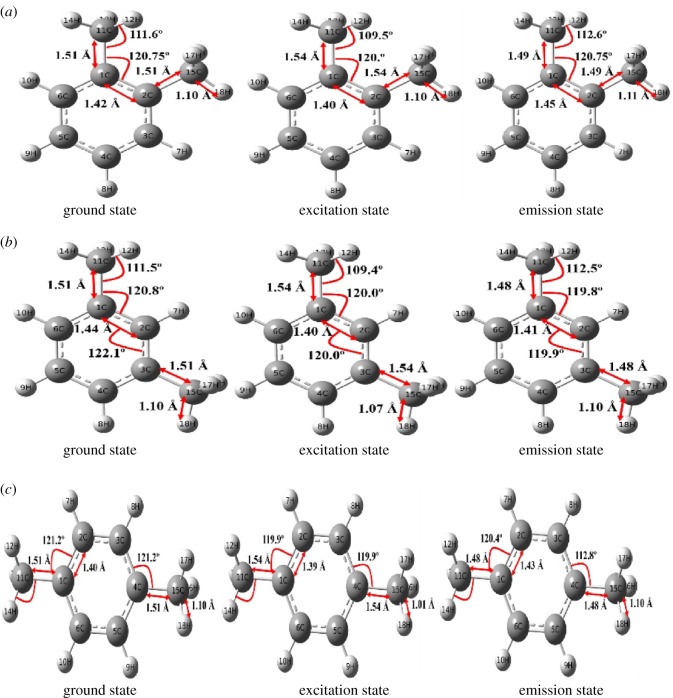
Bond energy and bond angles at methyl substitution position during fluorescence excitation and emission process: (*a*) *o*-xylene, (*b*) *m*-xylene and (*c*) *p*-xylene.

In this study, the latest revision of the Gaussian 09 program package [28] was used for all TD-DFT calculations [41,42]. To optimize the ground state geometry of xylene isomers, the PBEPBE functional [29,30] together with the 6-31 + G (d, p) basis set [31] was employed, which has been used previously for different organic compounds and verified to give certain geometrical structures and normal mode frequencies [43]. All stationary points were confirmed as energy minima in the potential energy using vibrational frequency calculations. The ground state transition energies of xylenes in water were obtained by optimizing the geometry using DFT in conjunction with PBEPBE, whereas the excited state geometry was optimized using TD-DFT, with the PBEPBE functional and the 6-31 + G (d, p) basis set [26]. With the optimized ground state and excited state geometries, the absorption and emission spectra of the xylene isomers were calculated using the TD-DFT method with the PBEPBE functional and the 6-31 + G(d, p) basis set. The solvent effect on the transition energies was calculated by using the CPCM of the self-consistent reaction field theory [42–44]. The quantum mechanics study explains the changes in geometry, bond angle and bond length during optimization from the ground state to the excited state. These changes provide information about molecular excitation activity and reactivity of molecules in a solvent. To measure the reactivity of molecules, the softness of these molecules was calculated (electronic supplementary material, S2). The reactivity indices in terms of softness show a good agreement with the experimental reactivity in terms of fluorescence. The reactivity of *p*-xylene is highest when compared with that of the other two isomers, and the order of reactivity was the same as that of the experimental data, that is *p*-xylene > *o*-xylene > *m*-xylene. This difference of reactivity is due to the structural difference of these isomer molecules. The molecular structure of *o*-xylene possesses two methyl groups at adjacent carbons of the benzene ring; the angle of methyl carbon with the benzene ring stretches in excited state geometry. The bond length between methyl carbon and benzene ring carbon expands from 151 to 154 Å and shrinks again at the optimization state from 154 to 149 Å in *o*-xylene. Although the structure of *m*-xylene is different from that of *o*-xylene, the values of bond angles and bond lengths of methyl groups are almost the same as those of *o*-xylene (figure 6). The *p*-xylene possesses methyl groups as substituents at opposite corners of the benzene ring, and the values of bond length of the methyl group with the benzene ring stretch in the excited state and then shrink at optimization. The bond angle between the methyl substituent in *p*-xylene is 121° in the ground state and 119° in the excited state, and at the optimization state, the bond angle reduces to 112°. The bond angle of the methyl substituent with the benzene ring in *p*-xylene has a significant difference in values when compared with those of *o*- and *m*-xylene. These structural changes indicate that *p*-xylene has higher molecular activity and rotation of bond angles than *o*- and *m*-xylene. The state transition electric dipole moments of these molecules at the excited and emitted state explain the reactivity indices. Therefore, the interconversion energy value is higher for *p*-xylene than for the other two isomers. According to these results, the molecular activity of *p*-xylene is the reason for the lower excitation energy to excite *p*-xylene, its longer excitation/emission wavelength, higher experimental fluorescence intensity and more active interaction in the mixture.

The fluorescence intensities in binary mixture and ternary mixture also appeared to be different from those of individual isomers in water. The binary mixture of *o*-, *m*-xylene demonstrates lower quantum yield than *o*-, *p*-xylene and *m*-, *p*-xylene. This is due to the molecular interaction of isomer molecules with each other and water (solvent). The difference in fluorescence intensity increases with higher concentration in water. The mixed isomers in water have interactions between each other. The deviation of the predicted value is significant in a binary mixture of *o*-, *m*-xylene and the ternary mixture of the isomers. Similarly, the addition of *p*-xylene influences the fluorescence intensity in the mixture with the other isomers. It has been observed that the fluorescence intensity is a useful tool to differentiate the isomers in mixtures with a constant ratio. This study provides a brief insight on fluorescence properties and shows a way forward to use fluorescence intensity as a potential tool for a quantitative analytical method to characterize xylene isomers in aqueous solution. The fluorescence intensity at the same concentration was influenced by the addition of *p*-xylene to other isomers, as it possesses the highest fluorescence intensity among the three isomers. These findings indicate that the sole xylene isomers could be identified easily based on fluorescence peak location and correlation between fluorescence intensity and concentration. The mixture of xylenes (widely used as a solvent) has a linear correlation to the concentration only if the ratios of the isomers remain constant. It is further noted that there exists interaction among the xylene isomer molecules in water, which leads to deviation of fluorescence intensities of isomers in mixtures. *p*-Xylene seems to be able to exhibit the strongest interaction. The higher intensity of *p*-xylene is due to the nonlinear molecular geometry of the molecule, originated from more delocalized conjugated π electrons over the molecule when compared with the other isomers.

#### 3.3.1. Excitation

**Figure 7.**
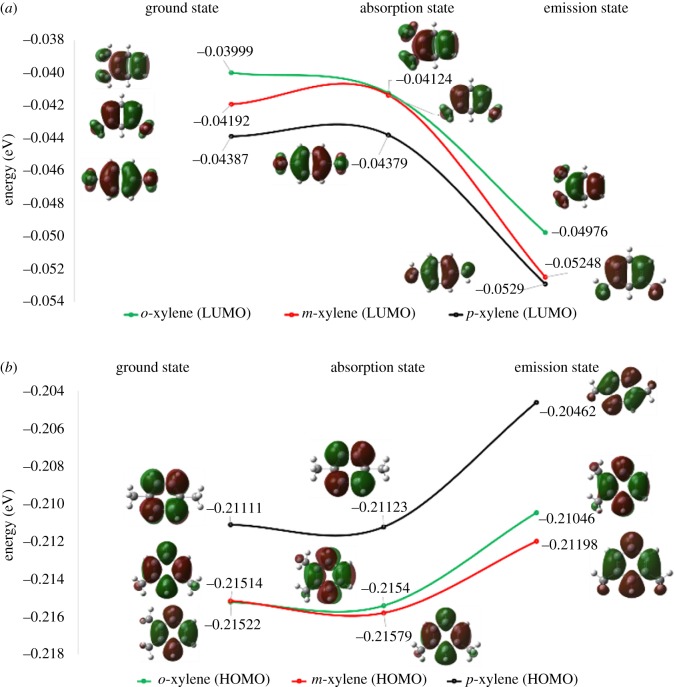
The molecular orbital energy and some contours of molecular orbitals in ground, absorption and emission states: (*a*) LUMO and (*b*) HOMO.

To understand the theoretical excitation and emission of xylene isomers, TD-DFT calculations were conducted. The absorption spectra of xylene isomers are calculated by TD-DFT/PBEPBE methods applying the 6-31 + G (d, p) basis set. TD-DFT is used to predict the excited state spectra for the fluorescence excitation of the compound. The calculated theoretical data of wavelengths, oscillator strengths and alpha molecular orbitals, highest energy occupied molecular orbitals (HOMOs) and lowest energy unoccupied molecular orbitals (LUMOs) [45] provide information about the molecular activity during the fluorescence process. The corresponding simulated ground state geometry and excited state geometry shapes, as distinctly shown with the HOMO and the LUMO, also explain the changes in an electronic cloud around the molecule [46] (figure 7).

Similarly, the molecular orbital contour energy (eV) of ground, absorption and emission states of *o*-, *m*- and *p*-xylene was calculated. The energy difference in LUMOs and HOMOs describes the molecular interaction as in the case of *p*-xylene; the energy difference is higher than for *o*- and *m*-xylene. The difference of excitation and emission energies was the energy used by the system for internal rearrangement of the molecule after photoexcitation. These data of interconversion transition energies are obtained by deducting the second-excited-state transition energy from the first-excited-state transition energy. The theoretical wavelength observed for excitation of *o*-xylene is 247 nm, for *m*-xylene 348 nm and for *p*-xylene 251 nm. The difference in excitation wavelength for *o*- and *m*-xylene is 1 nm, whereas for *p*-xylene the difference is 3–4 nm. As the EEM is measured under a slide length of 5 nm, the experimental excitation wavelength of *o*- and *m*-xylene is the same, and that of *p*-xylene is a 5 nm difference from those of *o*- and *m*-xylene. The oscillator strength for excited state transition for *o*-xylene is 0.0106, for *m*-xylene 0.005 and for *p*-xylene 0.133. The theoretical results of *p*-xylene indicated higher oscillator strength and longer wavelength compared to the other two isomers.

#### 3.3.2. Emission

The excited state geometry of xylene has been optimized by using the TD-DFT method, PBEPBE functional and 6-31 + G (d, p) basis set. The emission spectra were obtained by using the TD-DFT method, PBEPBE functional and 6-31 + G (d, p) basis set by the optimization of excited state geometries. The emission wavelength and its corresponding oscillator strength for xylene isomers are illustrated in table 1. The lowest emission wavelength was found for *o*-xylene at 267 nm, in comparison with *m*-xylene at 269 nm and *p*-xylene at 307 nm. The theoretical wavelengths of absorption spectra and emission spectra were also assigned in π–π* characterization from the HOMO to the LUMO [47,48] (figure 7). The Stokes shift for *p*-xylene was about 17 nm, whereas that for *o*- and *m*-xylene was 18 and 16 nm, respectively. The TD-DFT calculation describes more considerable rearrangement energy upon photoexcitation for the fluorescence of *p*-xylene. The influence of water as a solvent on the emission spectra of xylene isomers was also considered by using the CPCM. The theoretical emission wavelengths of the three isomers exhibit similar trends with regard to absorption spectra.

**Table 1.** Experimental and theoretical excitation and emission wavelengths.

compound	solvent	experimental excitation wavelength (nm)	theoretical excitation wavelength (nm)	alpha molecular orbitals	oscillator strength (*f*)	experimental emission wavelength (nm)	theoretical emission wavelength (nm)	alpha molecular orbitals	oscillator strength (*f*)
*o*-xylene	water	260	247	HOMO	0.0106	285	267	HOMO	0.0187
				(−0.22385)				(−0.33303)	
				LUMO				LUMO	
				(−0.21540)				(−0.16325)	
*m*-xylene	water	260	248	HOMO	0.005	285	269	HOMO	0.0175
				(−0.21579)				(−0.21198)	
				LUMO				LUMO	
				(−0.04136)				(−0.05348)	
*p*-xylene	water	265	251	HOMO	0.0133	290	307	HOMO	0.0339
				(−0.21123)				(−0.20462)	
				LUMO				LUMO	
				(−0.04379)				(−0.055290)	

The theoretical results provide necessary information about the individual photophysical properties of each isomer. The fluorescence properties of the three isomers show a significant difference. The theoretical emission oscillator strength of xylene isomers was found to be in line with experimental fluorescence intensity findings, in the order of *p*-xylene > *o*-xylene > *m*-xylene in water. This difference in properties is derived from the position of the substituent as the methyl groups of the xylene molecule are substituted at *ortho*, *meta* and *para* positions. The variation in the excited and emitted energies of *p*-xylene is greater than those of *o*- and *m*-xylene. Therefore, *p*-xylene possesses higher rearrangement energy than *m*-xylene and *o*-xylene, due to its molecular geometry and nonlinear structure. This finding could be used to distinguish xylene isomers in water as an easy and fast method.

#### 3.3.3. Interaction between isomers

The computational details about the energy change in the solution provide the proof about the interaction of molecules in the mixture, studied by optimizing the frequency of sole and mixture of isomers in the ground state (electronic supplementary material, S3). The thermal correction to the energy, thermal correction to enthalpy and thermal correction to Gibbs free energy show variations in the study of sole molecules and mixtures [49]. These energy variations were studied at ground state, and the results show good agreement with experimental results. The value of Gibbs free energy determines the fate of the reaction of compounds in solution, and whether the reaction is spontaneous or not spontaneous. According to the present results, all the reactions with sole and mixture molecules in aqueous solution are spontaneous as the value of Gibbs free energy remains below one. The difference in calculated thermal correction energy and thermal correction to Gibbs free energy shows the highest value for *p*-xylene. Therefore, the reactivity of *p*-xylene is highest when compared with that of *o*- and *m*-xylene and hence produces strongest experimental fluorescence signals with highest fluorescence intensity. The more spontaneous system was found for *p*-xylene at ground state than for *m*-xylene and *o*-xylene. Theoretically calculated thermal correction energy with Gibbs free energy was higher for the ternary mixture, whereas for the binary mixture the highest energy was observed for *m*- and *p*-xylene. These theoretical findings are in line with the experimental results.

## 4. Conclusion

The ground state geometries were fully optimized using the PBEPBE functional methods, in combination with the 6-31 + G(d,p) basis set. A frequency analysis at the same level of theory was used for each optimized structure, in the ground state. The excited state energies were calculated with TD-DFT, and a CPCM was employed to study the solvent effect. The fluorescence technique combined with TD-DFT can be used as a new quantitative method for the identification of sole and mixed xylenes at constant ratios in water solution. The slope of fluorescence intensity versus concentration curve is a very good parameter to detect the xylene isomers in solution. The fluorescence intensity, peak location and molecular interactions in mixtures provide significant information to distinguish isomers. Therefore, the fluorescence approach is a suitable method to detect the pollution of xylene in solutions. The theoretical results elaborate the energy behaviour of the compounds and indicate a substantial agreement with experimental findings.

## Supplementary Material

S1-Computational details of the molecular dynamics simulation and reactivity indices; S2 Molecular Softness; S3-Computational details of molecular interaction

## Data accessibility

The datasets supporting this article have been uploaded as part of the electronic supplementary material, and at: http://dx.doi.org/10.5061/dryad.3s06p [50].
