# Supplementary material for: Fluorescence and photophysical properties of xylene isomers in water: with experimental and theoretical approaches
Source: R Soc Open Sci. 2018 Feb 7;5(2):171719. doi: 10.1098/rsos.171719 (PMC5830770; doi:10.1098/rsos.171719)
Supplement: S1-Computational details of the molecular dynamics simulation and reactivity indices; S2 Molecular Softness; S3-Computational details of molecular interaction [file rsos171719supp1.docx]

# **Fluorescence and Photophysical Properties of Xylene Isomers in Water: with Experimental and Theoretical Approaches**

**Supporting Information**

MUHAMMAD FAROOQ SALEEM KHAN^†^, JING WU*†, BO LIU^†^, CHENG CHENG^†^, MONA AKBAR^†^ ,YIDI CHAI^†^, AISHA MEMON^†^,

^†^State Key Joint Laboratory of Environment Simulation and Pollution Control, School of Environment, Tsinghua University, Beijing 100084, China.

**Corresponding author:**

^*^JING WU (E-mail: [wu_jing@tsinghua.edu.cn](mailto:wu_jing@tsinghua.edu.cn). Phone: +86-10-62789121. Fax: +86-10-62785687)

# **Contents**

1. S1- Computational details of the molecular dynamics simulation and reactivity indices
2. S2- Molecular softness
3. S3- Computational details of molecular interaction

# **S1 Computational details of the molecular dynamics simulation**

The quantum mechanics of molecular dynamics simulation was carried out on the solvated chemicals using the Gaussian 9 software package^1^. With the aid of the Gauss view software, an initial geometry was generated for single molecule by CPCM solvation as water molecules. All DFT calculations are performed with the Gaussian, with 6-31+G (d, p) basis set and using PBEPBE functional ^1-5^. The excitation and emission wavelengths were recorded and the detail of reactivity of molecules at excited, and emission state described in as below;

## **1.1 Reactivity indices**

### **1.1.1 o-Xylene**

1. **o-xylene (Excitation):**

Ground to excited state transition electric dipole moments (Au):

State X Y Z Dip. S. Osc.

1 0.1499 0.0000 0.0000 0.0225 0.0029

2 0.0000 0.3761 0.0001 0.1414 0.0201

3 0.0000 0.0002 0.2431 0.0591 0.0088

4 0.0005 0.0000 0.0000 0.0000 0.0000

5 0.0000 -0.0005 0.0878 0.0077 0.0012

6 0.0001 0.0000 0.0000 0.0000 0.0000

Ground to excited state transition velocity dipole moments (Au):

State X Y Z Dip. S. Osc.

1 -0.0263 0.0000 0.0000 0.0007 0.0024

2 0.0000 -0.0786 0.0000 0.0062 0.0193

3 0.0000 0.0000 -0.0412 0.0017 0.0051

4 -0.0001 0.0000 0.0000 0.0000 0.0000

5 0.0000 0.0001 -0.0174 0.0003 0.0009

6 0.0000 0.0000 0.0000 0.0000 0.0000

Ground to excited state transition magnetic dipole moments (Au):

State X Y Z

1 -0.0001 0.0000 0.0000

2 0.0000 0.0000 0.2296

3 0.0000 -0.2158 0.0001

4 0.5225 0.0000 0.0000

5 0.0000 -0.4981 -0.0002

6 0.1138 0.0000 0.0000

1. **o-Xylene (Emission) :**

Ground to excited state transition electric dipole moments (Au):

State X Y Z Dip. S. Osc.

1 0.4114 0.0000 0.0000 0.1692 0.0208

2 0.0000 0.1195 -0.0442 0.0162 0.0023

3 0.0000 0.0656 -0.2487 0.0661 0.0094

4 0.0000 2.2716 0.0212 5.1607 0.7593

5 -0.1597 -0.0001 0.0000 0.0255 0.0038

6 0.0000 0.3048 -0.1412 0.1128 0.0168

7 -2.5488 0.0000 0.0000 6.4964 0.9722

8 0.1237 0.0000 0.0000 0.0153 0.0023

9 -0.1646 0.0000 0.0000 0.0271 0.0042

10 0.0000 0.0905 -0.4030 0.1706 0.0270

Ground to excited state transition velocity dipole moments (Au):

State X Y Z Dip. S. Osc.

1 -0.0755 0.0000 0.0000 0.0057 0.0207

2 0.0000 -0.0248 0.0091 0.0007 0.0022

3 0.0000 -0.0143 0.0418 0.0020 0.0061

4 0.0000 -0.4903 -0.0039 0.2404 0.7263

5 0.0350 0.0000 0.0000 0.0012 0.0037

6 0.0000 -0.0660 0.0267 0.0051 0.0151

7 0.5603 0.0000 0.0000 0.3139 0.9323

8 -0.0282 0.0000 0.0000 0.0008 0.0023

9 0.0379 0.0000 0.0000 0.0014 0.0041

10 0.0000 -0.0209 0.0914 0.0088 0.0247

Ground to excited state transition magnetic dipole moments (Au):

State X Y Z

1 -0.0032 0.0000 0.0000

2 0.0000 -0.0480 -0.0228

3 0.0000 0.1858 0.0274

4 0.0000 -0.0636 1.0428

5 0.4873 0.0000 0.0000

6 0.0000 0.4553 0.1741

7 -0.0051 0.0000 0.0000

8 0.0934 0.0000 0.0000

9 0.2354 0.0000 0.0000

1. 0.0000 0.3064 -0.0183

### **1.1.2 m-Xylene**

1. **m-xylene (Excitation):**

Ground to excited state transition electric dipole moments (Au):

State X Y Z Dip. S. Osc.

1 0.2025 0.0000 0.0071 0.0410 0.0050

2 0.0000 0.2641 0.0000 0.0698 0.0097

3 0.0000 0.0329 0.0000 0.0011 0.0002

4 0.1393 0.0000 -0.1210 0.0341 0.0050

5 0.0000 -1.7913 0.0000 3.2088 0.4853

6 0.4007 0.0000 0.1165 0.1741 0.0266

Ground to excited state transition velocity dipole moments (Au):

State X Y Z Dip. S. Osc.

1 -0.0382 0.0000 -0.0013 0.0015 0.0053

2 0.0000 -0.0547 0.0000 0.0030 0.0096

3 0.0000 -0.0063 0.0000 0.0000 0.0001

4 -0.0306 0.0000 0.0203 0.0013 0.0041

5 0.0000 0.3962 0.0000 0.1570 0.4614

6 -0.0884 0.0000 -0.0288 0.0086 0.0251

Ground to excited state transition magnetic dipole moments (Au):

State X Y Z

1 -0.0149 0.0000 0.3978

2 0.0000 -0.0428 0.0000

3 0.0000 -0.1523 0.0000

4 0.4489 0.0000 0.0797

5 0.0000 -0.0312 0.0000

6 -0.3119 0.0000 0.0824

1. **m-Xylene (Emission) :**

Ground to excited state transition electric dipole moments (Au):

State X Y Z Dip. S. Osc.

1 0.3248 0.0000 0.0060 0.1055 0.0134

2 0.0000 0.2551 0.0000 0.0651 0.0092

3 0.0000 0.3720 0.0000 0.1384 0.0205

4 0.0000 -2.2460 0.0000 5.0444 0.7539

5 2.4904 0.0000 -0.0007 6.2023 0.9444

6 -0.7408 0.0000 -0.1681 0.5770 0.0885

Ground to excited state transition velocity dipole moments (Au):

State X Y Z Dip. S. Osc.

1 -0.0625 0.0000 -0.0012 0.0039 0.0137

2 0.0000 -0.0541 0.0000 0.0029 0.0092

3 0.0000 -0.0798 0.0000 0.0064 0.0191

4 0.0000 0.4904 0.0000 0.2405 0.7151

5 -0.5564 0.0000 -0.0018 0.3096 0.9036

6 0.1665 0.0000 0.0318 0.0287 0.0833

Ground to excited state transition magnetic dipole moments (Au):

State X Y Z

1 -0.0144 0.0000 0.4123

2 0.0000 -0.0407 0.0000

3 0.0000 -0.2736 0.0000

4 0.0000 -0.0805 0.0000

5 0.1259 0.0000 0.6406

1. 0.5973 0.0000 -0.1546

### **1.1.3 p-Xylene**

1. **p-xylene (Excitation) :**

Ground to excited state transition electric dipole moments (Au):

state X Y Z Dip. S. Osc.

1 0.0000 -0.2572 0.0000 0.0661 0.0080

2 0.0000 0.0000 0.8498 0.7221 0.0987

3 0.0000 0.0000 -0.1121 0.0126 0.0018

4 -0.0163 0.0000 0.0000 0.0003 0.0000

5 0.0000 -1.0011 0.0000 1.0021 0.1508

6 0.0000 1.3056 0.0000 1.7045 0.2579

7 0.0000 0.0000 0.0000 0.0000 0.0000

8 0.0000 0.0000 -2.1496 4.6210 0.7214

9 0.0000 0.0000 0.0000 0.0000 0.0000

10 0.0000 0.0000 -0.5170 0.2673 0.0424

Ground to excited state transition velocity dipole moments (Au):

State X Y Z Dip. S. Osc.

1 0.0000 0.0487 0.0000 0.0024 0.0087

2 0.0000 0.0000 -0.1731 0.0300 0.0974

3 0.0000 0.0000 0.0249 0.0006 0.0020

4 -0.0013 0.0000 0.0000 0.0000 0.0000

5 0.0000 0.2193 0.0000 0.0481 0.1421

6 0.0000 -0.2867 0.0000 0.0822 0.2414

7 0.0000 0.0000 0.0000 0.0000 0.0000

8 0.0000 0.0000 0.4961 0.2462 0.7008

9 0.0000 0.0000 0.0000 0.0000 0.0000

10 0.0000 0.0000 0.1200 0.0144 0.0404

Ground to excited state transition magnetic dipole moments (Au):

State X Y Z

1 0.0000 0.0000 -0.0392

2 0.0000 -0.0734 0.0000

3 0.0000 0.0272 0.0000

4 0.0000 0.0000 0.0000

5 0.0000 0.0000 0.3894

6 0.0000 0.0000 0.4962

7 0.1041 0.0000 0.0000

8 0.0000 0.1331 0.0000

9 0.0376 0.0000 0.0000

10 0.0000 -0.3577 0.0000

1. **p-Xylene (Emission) :**

Ground to excited state transition electric dipole moments (Au):

State X Y Z Dip. S. Osc.

1 0.0000 -0.4017 0.0000 0.1614 0.0196

2 0.0000 0.0000 1.1385 1.2961 0.1758

3 0.0000 0.0000 -0.0965 0.0093 0.0013

4 0.0000 2.0190 0.0000 4.0764 0.5932

5 0.0010 0.0000 0.0000 0.0000 0.0000

6 0.0000 0.1659 0.0000 0.0275 0.0041

7 0.0000 0.0000 -2.5439 6.4713 0.9736

8 0.0000 0.0000 0.0000 0.0000 0.0000

9 0.0000 0.0000 0.0000 0.0000 0.0000

10 0.0000 0.0000 -0.2190 0.0479 0.0076

Ground to excited state transition velocity dipole moments (Au):

State X Y Z Dip. S. Osc.

1 0.0000 0.0742 0.0000 0.0055 0.0202

2 0.0000 0.0000 -0.2296 0.0527 0.1727

3 0.0000 0.0000 0.0215 0.0005 0.0015

4 0.0000 -0.4282 0.0000 0.1834 0.5601

5 -0.0049 0.0000 0.0000 0.0000 0.0001

6 0.0000 -0.0358 0.0000 0.0013 0.0038

7 0.0000 0.0000 0.5652 0.3194 0.9437

8 0.0000 0.0000 0.0000 0.0000 0.0000

9 0.0000 0.0000 0.0000 0.0000 0.0000

10 0.0000 0.0000 0.0501 0.0025 0.0070

Ground to excited state transition magnetic dipole moments (Au):

State X Y Z

1 0.0000 0.0000 -0.0415

2 0.0000 -0.0929 0.0000

3 0.0000 0.0095 0.0000

4 0.0000 0.0000 0.0877

5 0.0000 0.0000 0.0000

6 0.0000 0.0000 0.6680

7 0.0000 0.0774 0.0000

8 0.1106 0.0000 0.0000

9 0.0384 0.0000 0.0000

10 0.0000 -0.3916 0.0000

## **S2 Molecular Softness**

To further analyze the difference between the xylene isomers (o-,m-, and p-xylene), molecular softnesses were calculated. The study of softness of molecules is used to measure its reactivity and are calculated using formula (1) ^6^.

$S=\frac{1}{E (LUMO) - E (HOMO)}$ (1)

Where *E* (LUMO) and *E* (HOMO) are the calculated energy of the lowest unoccupied molecular orbital and highest occupied molecular orbital respectively.

Table-1: Molecular softness of xylenes (1/a.u.).

| **Compound** | **Softness** |
| --- | --- |
| o-xylene | 5.7 |
| m-xylene | 5.5 |
| p-xylene | 6 |

The table-1 above shows the softnesses of xylene isomers. The softness result give the information about the presence of good reactivity indeices by these molecules in solvent. The difference between all three isomers is not very large but it appeared to be very significant as it give good agreement to the experimental reactivity in terms of fluorescence intensity. Such as the p-xylene posesses highest reactivity as compared to o-, and m-xylene, with order;

p-xylene>o-xylene>m-xylene.

## **S3 Computational details of molecular interaction**

The total thermal correction energy , thermal correction to enthalpy and thermal correction Gibb’s free energy was abtained by optimizing frequency at ground state was studied by applying PBEPBE basis with DFT and CPCM. The results indicate the energy difference of sole molecular systems and mixture molecular systems are in line with the interaction identified through experimental findings. According to results, the p-xylene poses more effect to the system on mixing with other isomers in binary and ternary mixtures. Similarly, the difference of thermal correction energy and Gibb’s free energy has been found highest for p-xylene in sole molecular system and possess consequent effect on mixing with other molecules. The table-2 below explains the energies of the system study as sole molecules in water and in mixture molecules in water (binary and ternary).

**Table-2:** Reaction thermal energies, enthalpies and reaction Gibb’s free energy calculated at Ground state with PBEPBE and DFT

| **Molecule** | **System** | **Thermal correction to Energy** | **Thermal correction to Enthalpy**  **(ΔH)** | **Thermal Correction to Gibb’s free energy**  **(ΔG)** | **Difference in thermal correction energy and Gibb’s free energy** | **Dipole Moment (Debye)** |
| --- | --- | --- | --- | --- | --- | --- |
| o-xylene | Sole molecule | 0.163016 | 0.163960 | 0.123946 | 0.03907 | 1.0841 |
| m-xylene | Sole molecule | 0.160226 | 0.161170 | 0.120497 | 0.039729 | 0.4684 |
| p-xylene | Sole molecule | 0.158066 | 0.159010 | 0.118017 | 0.40049 | 0.1263 |
| o,m-xylene | Binary mixture | 0.325390 | 0.326334 | 0.252814 | 0.072576 | 0.5376 |
| m,p-xylene | Binary mixture | 0.325275 | 0.326219 | 0.247847 | 0.077428 | 0.5305 |
| o,p-xylene | Binary mixture | 0.323424 | 0.324368 | 0.256890 | 0.066534 | 0.9417 |
| o,m,p-xylene | Ternary mixture | 0.486547 | 0.487491 | 0.392690 | 0.093857 | 1.3498 |

Reference:

1. G. W. T. M. J. Frisch, H. B. Schlegel, G. E. Scuseria, M. A. Robb, J. R. Cheeseman, G. Scalmani, V. Barone, G. A. Petersson, H. Nakatsuji, X. Li, M. Caricato, A. Marenich, J. Bloino, B. G. Janesko, R. Gomperts, B. Mennucci, H. P. Hratchian, J. V. Ortiz, A. F. Izmaylov, J. L. Sonnenberg, D. Williams-Young, F. Ding, F. Lipparini, F. Egidi, J. Goings, B. Peng, A. Petrone, T. Henderson, D. Ranasinghe, V. G. Zakrzewski, J. Gao, N. Rega, G. Zheng, W. Liang, M. Hada, M. Ehara, K. Toyota, R. Fukuda, J. Hasegawa, M. Ishida, T. Nakajima, Y. Honda, O. Kitao, H. Nakai, T. Vreven, K. Throssell, J. A. Montgomery, Jr., J. E. Peralta, F. Ogliaro, M. Bearpark, J. J. Heyd, E. Brothers, K. N. Kudin, V. N. Staroverov, T. Keith, R. Kobayashi, J. Normand, K. Raghavachari, A. Rendell, J. C. Burant, S. S. Iyengar, J. Tomasi, M. Cossi, J. M. Millam, M. Klene, C. Adamo, R. Cammi, J. W. Ochterski, R. L. Martin, K. Morokuma, O. Farkas, J. B. Foresman, and D. J. Fox, 2009.

2. J. P. Perdew, K. Burke and M. Ernzerhof, *Physical Review Letters*, 1997, **78**, 1396-1396.

3. L. M. Roch and K. K. Baldridge, *Phys Chem Chem Phys*, 2017, **19**, 26191-26200.

4. A. M. Al-Soliemy, O. I. Osman, M. A. Hussein, A. M. Asiri and S. A. El-Daly, *J Fluoresc*, 2016, **26**, 1199-1209.

5. S. Fleming, A. Mills and T. Tuttle, *Beilstein J Org Chem*, 2011, **7**, 432-441.

6. b. Thierry De Meyera, Karen Hemelsoetb, Veronique Van Speybroeckb,∗, Karen De and Clercka, DOI: <https://doi.org/10.1016/j.dyepig.2013.10.048>.
